# Successfully integrating aged care services: a review of the evidence and tools emerging from a long-term care program

**DOI:** 10.5334/ijic.963

**Published:** 2013-02-22

**Authors:** Michael J. Stewart, Andrew Georgiou, Johanna I. Westbrook

**Affiliations:** Centre for Health Systems and Safety Research, Australian Institute of Health Innovation, University of New South Wales, Sydney, Australia; Centre for Health Systems and Safety Research, Australian Institute of Health Innovation, University of New South Wales, Sydney, Australia; Centre for Health Systems and Safety Research, Australian Institute of Health Innovation, University of New South Wales, Sydney, Australia

**Keywords:** integrated care, aged care, integrated delivery systems, literature review, PRISMA, Canada

## Abstract

**Background:**

Providing efficient and effective aged care services is one of the greatest public policy concerns currently facing governments. Increasing the integration of care services has the potential to provide many benefits including increased access, promoting greater efficiency, and improving care outcomes. There is little research, however, investigating how integrated aged care can be successfully achieved. The PRISMA (Program of Research to Integrate Services for the Maintenance of Autonomy) project, from Quebec, Canada, is one of the most systematic and sustained bodies of research investigating the translation and outcomes of an integrated care policy into practice. The PRISMA research program has run since 1988, yet there has been no independent systematic review of this work to draw out the lessons learnt.

**Methods:**

Narrative review of all literature emanating from the PRISMA project between 1988 and 2012. Researchers accessed an online list of all published papers from the program website. The reference lists of papers were hand searched to identify additional literature. Finally, Medline, Pubmed, EMBASE and Google Scholar indexing databases were searched using key terms and author names. Results were extracted into specially designed spread sheets for analysis.

**Results:**

Forty-five journal articles and two books authored or co-authored by the PRISMA team were identified. Research was primarily concerned with: the design, development and validation of screening and assessment tools; and results generated from their application. Both quasi-experimental and cross sectional analytic designs were used extensively. Contextually appropriate expert opinion was obtained using variations on the Delphi Method. Literature analysis revealed the structures, processes and outcomes which underpinned the implementation. PRISMA provides evidence that integrating care for older persons is beneficial to individuals through reducing incidence of functional decline and handicap levels, and improving feelings of empowerment and satisfaction with care provided. The research also demonstrated benefits to the health system, including a more appropriate use of emergency rooms, and decreased consultations with medical specialists.

**Discussion:**

Reviewing the body of research reveals the importance of both designing programs with an eye to local context, and building in flexibility allowing the program to be adapted to changing circumstances. Creating partnerships between policy designers, project implementers, and academic teams is an important element in achieving these goals. Partnerships are also valuable for achieving effective monitoring and evaluation, and support to ‘evidence-based’ policy-making processes. Despite a shared electronic health record being a key component of the service model, there was an under-investigation of the impact this technology on facilitating and enabling integration and the outcomes achieved.

**Conclusions:**

PRISMA provides evidence of the benefits that can arise from integrating care for older persons, particularly in terms of increased feelings of personal empowerment, and improved client satisfaction with the care provided. Taken alongside other integrated care experiments, PRISMA provides further evidentiary support to policy-makers pursuing integrated care programs. The scale and scope of the research body highlights the long-term and complex nature of program evaluations, but underscores the benefits of evaluation, review and subsequent adaptation of programs. The role of information technology in supporting integration of services is likely to substantially expand in the future and the potential this technology offers should be investigated and harnessed.

## Introduction

Providing efficient, effective and comprehensive care for older persons is one of the greatest policy issues currently facing governments [1–5]. Bodies such as the Organisation for Economic Cooperation and Development (OECD) and the Australian Productivity Commission have noted that the sector is stretched, fragmented, difficult to enter and navigate, suffers from service gaps, and has variations in quality [4, 6]. Older persons and their caregivers face major challenges accessing the system and coordinating disparate care providers which impacts on patient safety, outcomes, feelings of satisfaction with care, and overall health service efficiency [1–3].

There needs to be a re-evaluation of the way aged care services are provided, such as through increasing the integration of health and social service providers. Integrated care is defined as producing a “coherent set of methods and models on the funding, administrative, organisational, service delivery and clinical levels...[to] create connectivity, alignment and collaboration” [7, p. 3] between care providers, with the aim of improving outcomes for patients and other service users [7–9]. Integration in the aged care sector has been trialled in numerous international projects in the USA, Canada, the UK and continental Europe [8, 10–15]. These experiments have provided evidence that integrated care can: increase access to care; streamline existing care; promote more efficient use of existing resources; and improve the patient experience without increasing total service costs [3, 16–20].

Aware of these benefits, many governments are investigating policy options for greater integration of services [4, 8]. A recent literature review, however, concluded that there was a need for more research to understand how successful integrated care can be achieved [21].

Many of the previously mentioned experiments are small in scale, and very few of them have been comprehensively described or academically evaluated. One key exception to this is the PRISMA (Program of Research to Integrate Services for the Maintenance of Autonomy) project from Quebec, Canada. PRISMA is perhaps the most systematic and sustained body of research work in this area, with associated research spanning from 1988 [22] to 2011 [23]. A holistic evaluation of such a long-term experiment has the potential to provide valuable evidence and strengthen the bridge between research and public policy, something urgently needed [24]. Despite these needs, no independent systematic overview of this unique and sustained body of work has been undertaken.

This paper aims to address this gap. It first provides an overview of the PRISMA body of work, and synthesises the evidence in terms of structures developed, processes implemented, and outcomes demonstrated. Taking a holistic view of the PRISMA body of work, it then identifies themes running through the work that served to contribute to its success.

## Overview of the PRISMA project

PRISMA developed a coordination based model of integrated community care for older persons [25] aimed at coordinating hospital, respite, residential and community based care. Including a pilot phase, the program was implemented and evaluated over nine years in Quebec, Canada but has over 20 years of research related to program and tool development, and longer term evaluations.

Under the PRISMA model, all of the public, private and voluntary health and social service organisations responsible for caring for older people in a given area are coordinated under one umbrella organisation. Each individual service provider keeps their own structure and governance system, but agrees to work within the additional overarching structure. The aim was to have an integrated service delivery (ISD) network that did not replace existing structures, but to develop structures that were embedded within the existing system [26]. The model is an attempt at both vertical (between different levels e.g. linking of hospitals, residential facilities, and community care providers) and horizontal (e.g. across otherwise unconnected community care providers) integration [1].

PRISMA comprised six individual components: (a) coordination between decision-makers and managers at the regional and local levels; (b) single entry point; (c) single assessment instrument coupled with case-mix management system; (d) case management; (e) individualised service plan; and (f) computerised clinical chart. Implemented as a single system, the research project was designed to measure the impact of this particular model of integrated care on the health, satisfaction, and empowerment of frail older persons, their use of services, and the burden on the principal informal caregiver [26].

The success of the project informed a major reform of the Quebec health care and social services system [27] and has also been trialled in France.

## Method

This paper is a narrative review of literature related to the PRISMA project, focusing on articles authored or co-authored by members of the PRISMA team.

### Rationale

The PRISMA literature represents arguably the largest body of longitudinal evidence regarding service integration amongst a defined population. Whilst the PRISMA team have published their final empirical results [26, 27], these papers did not report on themes or lessons for researchers and policy-makers, nor did they extensively detail the project processes and structures. Thus this review was designed to provide a narrative account and appraisal of the entire body of PRISMA research evidence by a group external to the PRISMA researchers.

### Search strategy

We commenced by accessing the program website (www.prismaquebec.ca) and retrieving an online list of PRISMA articles. All English articles in the list (n=38) were identified. Two books self-published by the PRISMA team were also retrieved. The bibliographies of retrieved articles were hand searched for additional articles. Finally, Pubmed, Medline, EMBASE and Google Scholar indexing databases were searched using key author names and search terms including: ‘PRISMA’; ‘PRISMA Quebec’; and ‘aged care Quebec’.

A search was conducted to find additional academic and grey literature that mentioned the PRISMA project. The same indexing databases and key terms were used as above, in addition to a standard Google web search.

### Inclusion criteria

We included all articles authored or co-authored by members of the PRISMA team. We included articles that directly referenced the PRISMA Quebec study, the pre-PRISMA Bois-Francs pilot study, and the implementation of PRISMA in France. Also included were studies that were directly related to an aspect of the PRISMA work, even if this work pre-dated PRISMA or Bois-Francs. For example, papers detailing the development and adaptation of the SMAF (Functional Autonomy Measurement System) tool were included, even though this was developed more than 10 years before the PRISMA project began. This ensured that we had a complete overview of the full evolution of PRISMA.

Both empirical studies and opinion pieces were included to provide a richness of data. The search was limited to full length papers published in English.

### Data analysis

Data analysis methods evolved iteratively. Initially, data were extracted into a summative table which detailed study design, methodology, research questions, results, and relationship to the overall project. Key words, for example ‘tool’, ‘evaluation’, and ‘outcome of interest’ were allocated to each paper. An analysis of this table revealed high-level overarching themes which suggested the eventual structure/process/outcome analysis framework. As more specific themes emerged, data were transferred to more focused tables including tables dedicated to the screening and assessment tools, and tables which aggregated all relevant information for key outcomes of interest. Supplementary tables were used to condense information related to project processes, tool development, and empirical evidence of outcomes. Tables were developed in Excel. Each paper was given a separate row on the spread sheet but had common column headings. This allowed extraction of similar information for each paper and facilitated the identification of overarching themes and commonalities. The authors held regular team meetings to discuss themes and project direction. A log of meetings and analysis was kept and regularly updated.

## Results

### Overview of the research

Although the scale, scope and aims of the actual project are similar to many other experiments [8], the PRISMA research output is far and away greater than that produced by other teams. In total, 45 articles and two books authored or co-authored by members of the PRISMA team were accessed.

The wider search uncovered an additional 20 sources not authored by members of the team. Nine of these were reports developed by policy think-tanks, three were peer reviewed academic articles, two were PowerPoint slides of conference presentations, two were websites, two were submissions to Canadian government commissions and two were reports prepared by government departments.

PRISMA authored research was predominately concerned with the design, implementation and evaluation of the Bois-Francs pilot project and the main Quebec project. Four of the articles related to adaptation and implementation in France [15, 16, 28, 29], and one study [30] reported on evidence from both France and Quebec. The 45 articles accessed for this review can be broadly assigned to four categories (Table 1).

The grey literature referred almost exclusively to the Canadian arm of the project. In general, this literature discussed the concept of integration and used PRISMA as a one successful case study amongst others. None of the grey literature provided a thorough analysis of the project, its outcomes, or lessons able to be drawn.

The PRISMA team employed a variety of study types. The majority of studies were either cross-sectional or quasi-experimental, incorporating pre-testing, comparable groups, and multiple post-tests. Variations on the Delphi method, a qualitative design for gaining consensus amongst experts, were also used (Table 2).

### Structures, processes and outcomes underpinning implementation

The scope of the PRISMA work provides a unique opportunity to assess the entire life-cycle of an experimental integrated care project. Results could be categorised into three key areas: structures (how the project was organised), processes (how the project worked), and outcomes (what the project delivered). Processes and outcomes could further be divided into those related to the delivery of integrated aged care services, and those related specifically to research.

In this paper, outcomes are presented before processes. The tools designed by the project team, an outcome of the project, need to be explained before their use in processes can be detailed.

### Structures

The project established a tripartite governance structure which ran in parallel to existing individual service provider arrangements. Boards were established at the governance and service management levels, and a multidisciplinary advisory committee was established at the clinical level. In Quebec, a single board at the highest level provided common strategic and policy advice to the 3 intervention areas. Component implementation advice and timetabling was discussed at the regional/implementation level. At the local level, a multidisciplinary team provided clinical and managerial advice [26].

Committees included participants across a range of expertise including clinicians, change management experts, and local and regional government representatives. There was an emphasis on local knowledge and input, especially at the lower levels. Local collaboration with providers was encouraged. Academic teams provided guidance and evaluated the project, but were not specifically involved in implementation [26, 31, 60].

Existing structures were used to coordinate and deliver services. Case managers worked within existing local community health centres (CLSCs), and coordinated individual service providers but these providers were never amalgamated into a single organisation [26, 31]. The single entry point was linked to a pre-existing 24/7 health information telephone service [26].

The shared computerised clinical chart was one of the six core components of the PRISMA model. A chart was developed for the Bois-Francs pilot project [26], however full implementation was delayed due to technical limitations [27]. There was little detail in the literature of the components of the chart, nor of any other technological structures underpinning the project. There was also no comprehensive assessment of the role of technology in facilitating integration.

### Outcomes

*Service outcomes:* Table 3 details the service outcomes demonstrated by PRISMA. Nine primary outcomes of interest are presented in the table, including functional decline, service utilisation, and feelings of satisfaction and empowerment with care delivered. Both positive and negative/ results are presented, since results evolved over the course of the study. For example, there was no significant decrease in ‘functional decline’ over the first two years of the study, but a significant effect did emerge across all four years.

In summary, there was a reduction in both the incidence and prevalence of ‘functional decline’ amongst patients exposed to the PRISMA intervention, with an annual incidence of functional decline lower by 137 cases per 1000 individuals in the PRISMA group [26]. The program also demonstrated a progressive reduction in handicap levels [63], decreased prevalence of unmet needs for those living in the community [23, 26], and clients reported significantly improved feelings of satisfaction (p<0.001) [63] and empowerment (p<0.001) [26]. Feelings of satisfaction and empowerment increased as measured degrees of integration increased, providing additional evidence supporting the benefits arising from integrated care [26].

The program also demonstrated benefits to the wider health system. Older persons in the experimental group showed a more appropriate use of emergency rooms and health facilities. Whilst in general both groups consulted a family physician once a year, the proportion of clients in the PRISMA group consulting with a medical specialist once a year dropped from 60% to 50% (p<0.001). The comparative groups remained steady at 60% [63]. There was no demonstrated difference in rates of hospitalisations, length of stay, or readmissions, nor in use of home-care or volunteer services [63]. Whilst over the first 2 years of the project PRISMA demonstrated both a decreased desire to enter residential aged care facilities and significantly lower caregivers burden [9, 31], this was not maintained throughout the study.

The literature reported conflicting findings over the course of the study. For example, in older persons exposed to the intervention, the desire to be institutionalised (enter a residential aged care facility) significantly decreased at years one and two [31], but not across the four years of the study [26].

*Project outcomes:* The PRISMA project produced one of the most extensive evidence bases related to a single integrated care study. The scope and scale of the literature undoubtedly indicates a strong push to comprehensively assess, evaluate and publish.

Nineteen papers detailed the development and validation of the standardised tools, indicators and data collection methods. The research team designed and validated tools to assess all aspects of the project. Several tools (SMAF, PRISMA-7, Iso-SMAF profiles) were developed for the project, taking into account project context through designing, testing and modifying tools based on population and expert feedback. Previously existing tools including the Mini Mental State Examination (MMSE), Zarit Burden Interview, and Morycz Interview were tested for use in the project population, and adapted to context if needed [26]. Tools were used across the service process, and included tools for case finding and opportunistic screening (PRISMA-7), assessment of functional decline and disability (SMAF), and assessment of consumer empowerment and satisfaction. The project also produced unique and innovative means of assessment, such as the ‘degree of integration’ grid to quantify the perceived extent of integration amongst service providers [37].

The team undertook several qualitative studies examining aspects of project implementation [15, 42, 52]. The outcomes from these studies can be used to examine the feasibility of undertaking similar projects. The PRISMA team surveyed 127 family physicians, and found that respondents had a strong interest both in participating in integrated service delivery networks, and working with case managers. They did not appear to fear the intrusion of another professional into the physician-patient relationship [52]. In assessing the implementation of the PRISMA-France project [15], it was discovered that organisations appreciated the guidance of the experimental team, which enabled them to learn from each other and to develop inter-organisational relationships. However, this study also found a tendency by organisations to protect their individual identities.

In addition, the qualitative study of the PRISMA-France project [15] provides suggestions for future implementers. The team discovered that, at least for this project, the most committed change managers adopted a ‘help it happen’ approach, with strong top-down leadership. This was deemed to facilitate openness to local adaptation. In fact, respondents in this study requested stronger top-down leadership as they felt it would facilitate faster implementation [15].

Table 4 contains further detail on the suite of ten tools developed or adapted by the research team.

### Processes

**Figure 1:**
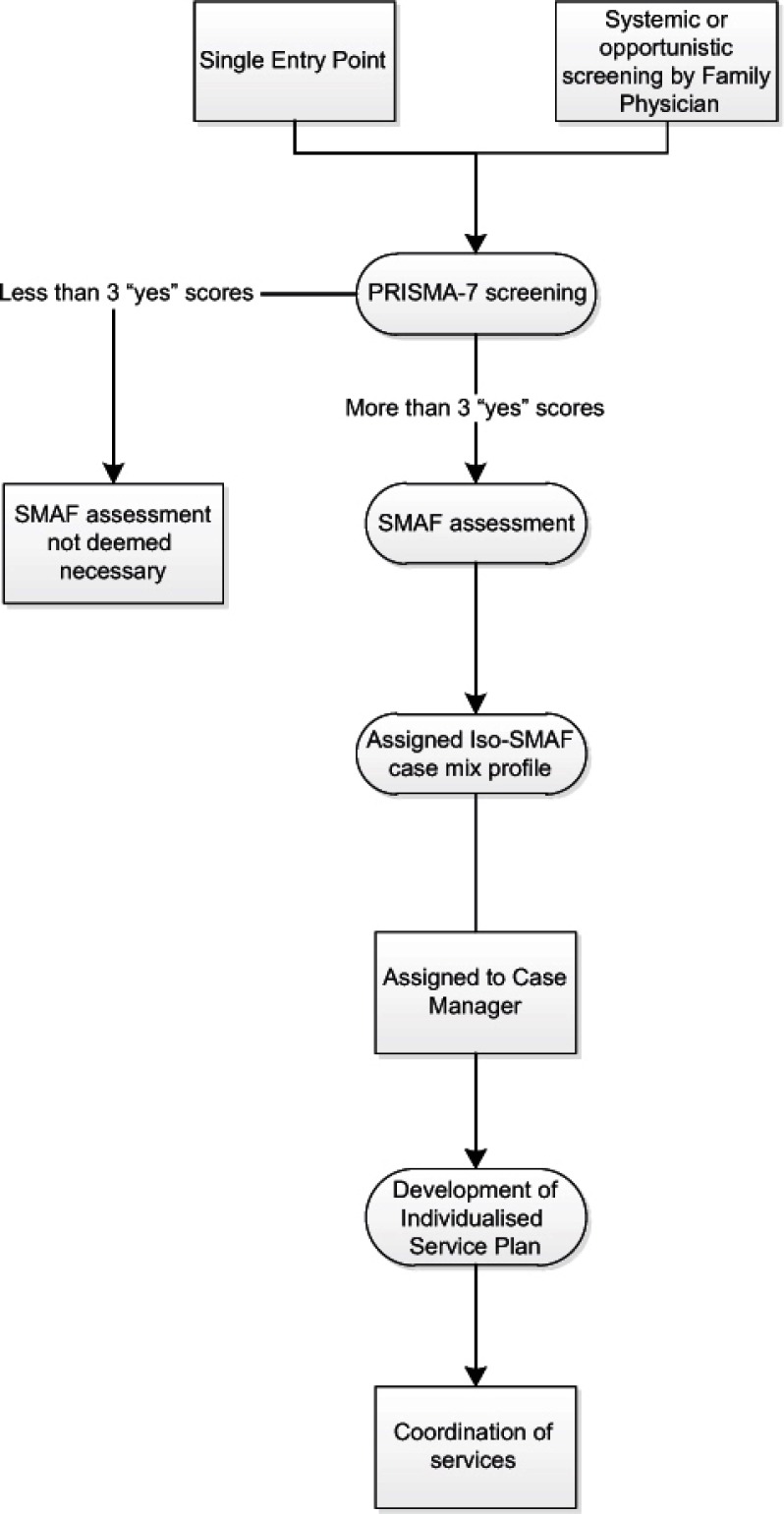
PRISMA screening and assessment process.

*Service processes:* A structured process for accessing services was developed (Figure 1). Prior to project entry, clients were screened for risk of functional decline using PRISMA-7. This was done by single entry point staff, or opportunistically by health care professionals who would onward refer [54]. Those deemed at risk of decline were then assessed with the SMAF and assigned an Iso-SMAF profile. A local case manager used the Iso-SMAF profile to coordinate care and resources. Coordination and provision of services was done on an as-needs basis [26]. Clients were reassessed with the SMAF every 12 months and assigned to a new Iso-SMAF profile as their needs changed.

PRISMA organised regular governance coordination meetings between national, regional and local stakeholders to plan project direction and provide service guidance [26].

*Project Processes:* PRISMA established a structured experimental process. A randomised controlled trial was considered unfeasible. Instead, a quasi-experimental design comparing 3 intervention areas with 3 control areas was chosen. Systematic methods were used to identify comparison areas. Clear inclusion criteria were defined and adhered to [26, 51, 56]. Although unable to recruit enough participants in year one, a matched ‘second wave’ cohort was enrolled in year three [26].

Participants were interviewed and assessed face-to-face pre-test and yearly for four years. Data on health and social services use was collected every two months by telephone. The reliability of data collection methods, such as assisted self-report and postal questionnaires, was assessed in pilot projects prior to use by the team [26, 31, 34, 41, 56, 63]. Every participant and their caregiver was given a calendar on which to record information, and trained accordingly [26]. This method showed strong reliability [41] and allowed for more complete information than using administrative data [26].

PRISMA used modified Delphi methods in which context appropriate tools were developed and validated by local experts. When choosing experts, the research team aimed to include views from all concerned stakeholder groups, include individuals with specific clinical or personal expertise, and to ensure representation of the diversity of geographic regions within Quebec [59]. A number of individual studies were nested within the overarching PRISMA design. Since large amounts of data were routinely collected, additional research questions could be answered as they arose [35, 41, 53, 55].

## Discussion

Analysing the PRISMA body of work reveals two key themes of interest to health policy-makers, project implementers, and researchers.

### The importance of context

Perhaps the key overarching theme which emerges from PRISMA is the effort made to design and adapt the project to its specific context. Adapting projects to context is neither new nor unique, but its importance cannot be overstated and is much discussed in literature. Internationally, there is an understanding that projects are influenced by external factors including national policy, funding arrangements, regulatory requirements [17, 22], and that no ‘off-the-shelf’ set of practices will lead to successful integration [22].

The PRISMA project designed structures and processes within the confines of its specific Quebecoise context. The program took advantage of structures that already existed, for example case managers worked through CLSCs, and the single entry point was attached to an existing telephone information hotline. Tools were specifically developed for the population of interest, providing a level of validity to the results obtained. When existing tools were used, they were tested for use with the project population, and then adapted if required.

The work provides a further push for governments and service providers to investigate home-grown solutions to policy problems. Building a program from the ground up, as occurred here, enables existing structures and conditions to be incorporated, and can help to limit unnecessary duplication and facilitate cost efficiencies. For example, attaching the entry point to an existing telephone hotline would have reduced project start-up costs. A similar project in Australia could attach itself to the already existing National Health Call Centre Network. Specifically designing tools for use with the population of interest provides a greater degree of validity to results achieved.

### Building in dynamism and adaptability

The second strength of PRISMA is its dynamism, adaptability, and acknowledgement that integration is an ongoing rather than one-off process [22]. The scale and scope of the research, as revealed through the structures and processes of the research portion of the project, provides evidence for this. More than just reporting demonstrated outcomes, the PRISMA team published the rationale behind certain data collection methods; the processes undertaken in developing the research; and the structures implemented to support the research. Nesting studies within the larger study framework allowed for the demonstration of more results than would otherwise have been expected. Whilst the results of the project would have been demonstrated regardless of these additional studies, the evident enthusiasm of the team was vital in disseminating and drawing attention to the research.

The project displayed the ability to adjust to changing circumstances. The team adapted to lower than expected numbers of enrolees in year one by setting up a second wave study in year three, making results comparable by ensuring the two cohorts were statistically similar. Measurement tools were consistently evaluated, validated and adapted if necessary. For example, the SMAF was initially developed in 1988 [22]. During the project it became evident that it failed to adequately measure the social functioning of older persons and a social new sub-scale, the social-SMAF, was developed and incorporated [30].

This aspect injected a degree of flexibility into the project. In large part, this appears to be the direct result of attaching an extensive and experienced research team to the project. The inclusion of an academic team brought the necessary expertise to enable the development of context specific tools, and to adapt these tools and indeed the whole project as circumstances required. A research team enables formative evaluations to occur, providing lessons learnt to feed back into the project with the developed of appropriate responses to fine tune the project. A qualitative evaluation of project implementation in France showed that organisations appreciated the support of a dedicated research team [15].

Specific funding for research teams, both at the design stage and for monitoring and evaluation purposes, is something that should be considered by policy-makers when they are designing integration projects. This could be a valuable strategy to increase project success and sustainability. There are many such calls for ‘evidence-based’ policy-making in the literature [67], and in particular the use of evidence emerging from reviews of qualitative and quantitative data [68]. The PRISMA project presents an example of how research run in parallel with service implementation can benefit service effectiveness, and advance the knowledge base for future service and policy developments.

## Assessment of the research

There are a few critiques that can be made of the PRISMA research. Even prior to implementation, health care service delivery in Quebec was more integrated than other systems [14]. The government was already the sole funder, manager and main provider of care. PRISMA researchers acknowledge that this may have diminished the ability of PRISMA to demonstrate a ‘value added’ effect [14]. It also suggests that in contexts with less baseline integration the outcomes achieved may be greater.

Despite the shared electronic health record being one of the six core PRISMA components, there was little discussion of its underlying technical structure, its implementation, or evaluation of its use. Information technology (IT) can be a major enabler to integrating care through facilitating the exchange of information between disparate care providers [42, 69]. The role that the shared PRISMA electronic health record played in facilitating integration and achieving the observed outcomes was under-investigated. Given the growing investments internationally in new information technologies it is clear they will play a prominent role in future health services [70], and may create new possibilities for supporting integration that will require research attention. For example, telehealth applications, email communication between health professionals and their clients, and in-home monitoring services are all growing modalities supporting the provision of care in different physical locations, and will lead to consumers interacting with their health professionals in different ways. Given that it is now well-known that IT systems can introduce new and unexpected behaviour changes, and be used in ways not initially envisioned [71], robust evaluations are needed to understand in much greater detail how IT can be used to support effective integration of care in innovative, safe, and efficient ways.

## Conclusions

The PRISMA body of work provides a valuable evidence base which supports policies which seek to increase the integration of aged care services. A sustained program of integration can decrease functional decline, and improve client satisfaction and empowerment with health care provided. Programs of integrated care should be strongly considered by governments and policy-makers.

The scale and scope of PRISMA makes clear that service evaluation is a long-term and complicated process that requires constant revision and adaption to changing circumstances. There is an evident benefit in fostering partnerships between academics, health policy-makers, and service planners and implementers. These partnerships should be encouraged, and actively supported by governments as an effective strategy by which to increase large-scale program effectiveness. The extent to which information technology in the form of a shared health record provided an enabling and supportive infrastructure in the PRISMA project remains under investigated. Health systems are increasingly investing in information technology (IT) as central infrastructure to efficient and effective care. Much greater research attention needs to be paid to the innovative ways in which IT may be a harnessed to achieve these service outcomes.

## Reviewers

**Kylza Aquino Estrellla,** Meducal Director, Hospitalar Santa Celina, Rua Conde de Lages, 44/403 Centro Rio de Janeiro RJ Brazil

**Jan Reed,** Emeritus Professor of Health Care for Older People, Northumbria University, UK

One anonymous reviewer

## Figures and Tables

**Table 1:**
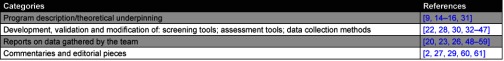
Overview of studies published by the PRISMA group

**Table 2:**
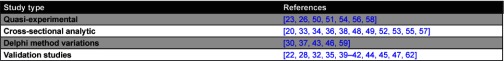
Study types employed by the PRISMA group

**Table 3:**
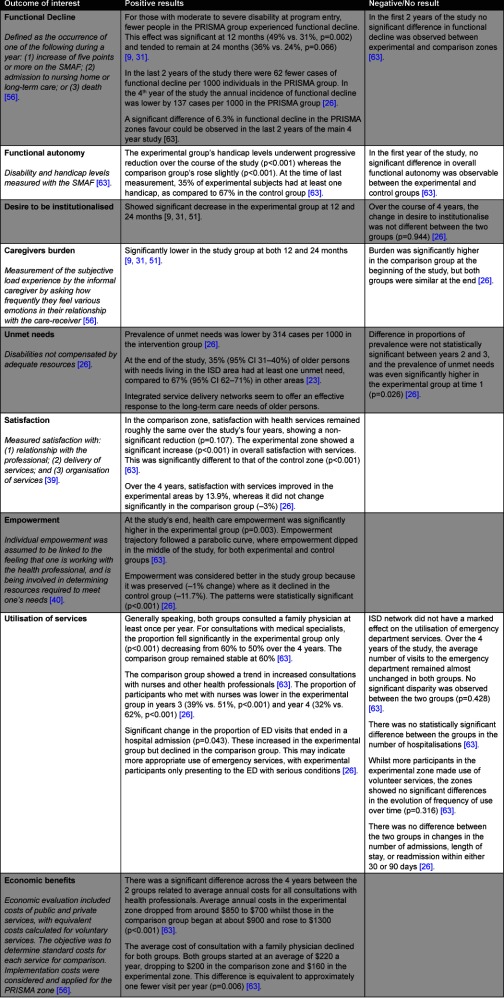
Detailed service outcomes from PRISMA integrated service delivery

**Table 4:**
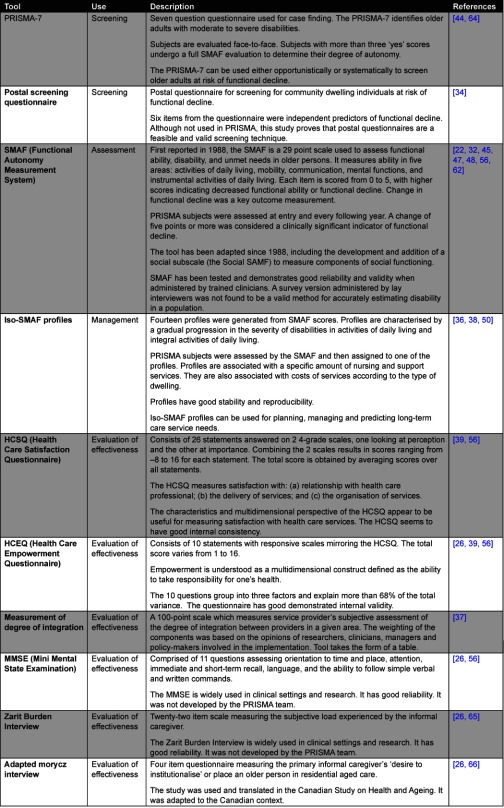
Detailed project outcomes from the PRISMA research work
